# Communicability of Diphtheria

**Published:** 1876-03

**Authors:** P. B. Pumyea

**Affiliations:** Imlaystown, N. J.


### Our Exchanges.

---

Communicability of Diphtheria.—*By P. B. Pumyea, M. D., Imlaystown, N. J.*—Is diphtheria contagious/ is a question now often asked the physician, and his reply is one of serious importance.

Were it not for the diversity of opinion among physicians as to the communicability of this extremely fatal disease, a statement of the following facts would be unnecessary. The history of the origin and progress of diphtheria, now in my practice, shows its contagious character, and how it *may* be propagated and restricted (in so far as a few cases can).

Charles D., and Mary K., cousins, were both servants in the same family. Mary K. was taken sick with sore throat, and went home. Although complaining a good deal, no physician was called until her only brother was taken sick with sore throat, and was hardly able to swallow. An inspection of his throat at once revealed the nature of the disease, and his subsequent death showed its malignancy. As the neighbors were very much alarmed, no one came near the house, except an uncle, who volunteered his services. He caught the disease and communicated it to his family. Three of his six children died.

On December 5th I was called to see Charles D., the cousin before mentioned, and ascertained that he had diphtheria. As he was not confined to his bed, and his employer did not wish to endanger his own family, he immediately took him home. Less than two weeks subsequently the disease appeared in that family. There were five boys and five girls, and as far as possible the girls were kept away from the boys. Not until all the boys had had the disease and recovered from it did any of the girls take it. They now have it, and one, the youngest, has died. The oldest girl did not live at home, but when she ascertained that her brothers were sick, she came home to visit them and thus caught the disease.

These three families live within a radius of half a mile, and there is not nor has not been another case of diphtheria within a radius of five miles.

Was not the disease in these cases communicated by contagion and its propagation restricted by isolation ?

We may not always be able to trace the origin and growth of this insidious disease. Sporadic cases undoubtedly occur as in cases of scarlatina. Sometimes the atmosphere seems to be so loaded with the contagious miasm as to produce an epidemic. But our inability to trace it or its epidemic character does not disprove its contagiousness, no more than it would that of pertussis or variola.

Prof. Smith, in his work on "Diseases of Children," says that the "communicability of this disease is as securely established as almost any doctrine in pathology."

It is much to be regretted that there is not *entire* unanimity of opinion among physicians in regard to the contagious nature of this disease, and that the people are not taught to shun it as they would small-pox.

When both the profession and the people acknowledge and appreciate the communicability of diphtheria, *then* it may be more restricted and our children better protected from this insatiable monster.

